# 1572. Impact of an Educational Initiative to Close Gaps in Knowledge, Confidence and Engagement in HIV Care

**DOI:** 10.1093/ofid/ofad500.1407

**Published:** 2023-11-27

**Authors:** Jason F Okulicz, Stephen J Fallon, J E W E L SAWYER, Jeffrey D Carter, Melissa Rodriguez, Shelbye Herbin, Laura Simone, Leah Molloy

**Affiliations:** San Antonio Military Medical Center, San Antonio, Texas; Latinos Salud, Wilton Manors, Florida; Avita Care Atlanta, Atlanta, Georgia; PRIME Education, LLC, Fort Lauderdale, Florida; PRIME Education, Fort Lauderdale, Florida; PRIME, Ferndale, Michigan; PRIME Education, LLC, Fort Lauderdale, Florida; PRIME Education, LLC, Fort Lauderdale, Florida

## Abstract

**Background:**

Ongoing engagement in HIV care is crucial to optimizing outcomes while addressing changing needs. This project aimed to identify and address gaps in HIV education, counseling and engagement.

**Methods:**

Between 12/2021 and 5/2022, 20 education sessions for people with HIV (PWH) were held in 5 US HIV clinics as a 4-part series emphasizing (1) benefits of viral suppression, (2) talking about sexual health, (3) HIV treatment options, and (4) aging well with HIV. Surveys were administered to participating PWH and healthcare professionals (HCP). Content was extended for nationwide use through an online toolkit for HCPs, and a peer mentorship video for PWH.

**Results:**

Surveys were completed by 312 PWH and 68 HCPs who participated in the live sessions. At baseline, 32% of HCPs were confident in counseling PWH about improving ART switching and 41% about adherence, which improved to 47% (p< 0.001) and 55% (p< 0.001), respectively, after accessing the online toolkit. Most PWH demonstrated greater knowledge about “undetectable = untransmittable” (96%) and the need for ART for all PWH regardless of viral load (86%) after the sessions than before (69%; p< 0.05 and 46%; p< 0.05, respectively). Compared to baseline, more HCPs reported high confidence in counseling PWH about achieving and maintaining viral suppression (96% vs 64%; p< 0.05) after the sessions. While not meeting the level of statistical significance, some increases in HCP confidence were identified in counseling PWH about coping with mental health challenges (90% vs 69%; p=0.34), and managing HIV with comorbidities (80% vs 58%; p=0.41). When asked about medication adherence, HCPs commonly identified health insurance (55%) as a barrier, while most PWH reported no barriers (61%). All participants identified top goals. Most PWH indicated a goal to educate themselves about HIV treatment and prevention while HCPs prioritized education and conversations about sexual wellness. As of August 2022, the online toolkit extension was accessed by 3,320 HCPs, and the peer mentorship video by 4,331 PWH.

**Conclusion:**

PWH knowledge and HCP confidence in counseling PWH improved following live collaborative educational sessions. Post-program goals were aligned amongst PWH and providers who prioritized improving PWH understanding of HIV management.

**Disclosures:**

**Jason F. Okulicz, MD**, Gilead Sciences: Advisor/Consultant **JEWEL SAWYER, PA-C, MSHS, AAHIVS**, Janssen: Speakers Bureau|Prime Education: Speakers Bureau

